# New Frontiers: Precise Editing of Allergen Genes Using CRISPR

**DOI:** 10.3389/falgy.2021.821107

**Published:** 2022-01-17

**Authors:** Nicole F. Brackett, Anna Pomés, Martin D. Chapman

**Affiliations:** INDOOR Biotechnologies Inc., Charlottesville, VA, United States

**Keywords:** CRISPR, gene editing, allergy, cat allergen, Fel d 1

## Abstract

Genome engineering with clustered regularly interspaced short palindromic repeats (CRISPR) technology offers the unique potential for unequivocally deleting allergen genes at the source. Compared to prior gene editing approaches, CRISPR boasts substantial improvements in editing efficiency, throughput, and precision. CRISPR has demonstrated success in several clinical applications such as sickle cell disease and β-thalassemia, and preliminary knockout studies of allergenic proteins using CRISPR editing show promise. Given the advantages of CRISPR, as well as specific DNA targets in the allergen genes, CRISPR gene editing is a viable approach for tackling allergy, which may lead to significant disease improvement. This review will highlight recent applications of CRISPR editing of allergens, particularly cat allergen Fel d 1, and will discuss the advantages and limitations of this approach compared to existing treatment options.

## Introduction

Allergic disease is a persistent clinical challenge with limited treatment options. Inhaled allergens, such as those derived from cat, pollen, or dust mite contribute to the development or exacerbation of IgE-mediated allergic rhinitis or asthma (1). While the prevalence of allergic rhinitis among young adults in developed countries has been found to range from 12 to 46% (2), treatment options are largely limited to allergen avoidance or medications that ease the allergic symptoms (e.g., antihistamines or corticosteroids). Targeted immunotherapies specific to pollen or dust mite allergens have recently proven effective for treating allergic rhinitis (3, 4). However, efficacy has not been demonstrated for many inhaled allergens and practical constraints such as treatment duration or expense limit the broad application of immunotherapy for the treatment of allergic rhinitis or asthma (1).

Food allergy may result in potentially fatal anaphylactic immune reactions and accounts for considerable annual healthcare costs (5). Previous studies indicate that an estimated 3–8% of children in the US suffer from food allergy, and suggest that the prevalence of food allergies has increased over time (6). Recent data support that early introduction to allergens is an effective strategy to prevent the onset of food allergy (7), whereas allergen avoidance may be necessary for disease management among sensitized individuals (8). Allergen immunotherapy strategies have been developed to prompt patient desensitization or tolerance in response to repeated food allergen exposure. Though allergen immunotherapy study data show promise, the requisite duration, safety, or maintenance of sustained immunotherapy treatments have not been fully elucidated (9).

Genome engineering, particularly CRISPR editing, offers the potential to effectively delete the allergen genes at the source, which may significantly benefit allergic individuals. This review will outline the advantages, limitations, and existing clinical applications of CRISPR technology. Several applications of CRISPR editing in allergy will be discussed, highlighting the value of the approach for engineering hypoallergenic food, developing allergen-free animal models, or determining the biologic function of allergen proteins. Though the therapeutic potential of CRISPR gene editing was only recently discovered, the technology will undoubtedly shape the evolution of disease management, and guide novel approaches for tackling allergic rhinitis or food allergy.

## CRISPR Gene Editing

### CRISPR Technology

The clustered regularly interspaced short palindromic repeats (CRISPR) systems function as a means of adaptive immunity in bacteria and archaea for the recognition and deletion of invading viral or plasmid DNA (10–14). CRISPR systems are comprised of small, modifiable guide RNAs (sgRNA) that direct CRISPR-associated (Cas) proteins to produce site-specific DNA double-stranded breaks (DSBs) (10). The well-known Cas9 nuclease, derived from *Streptococcus pyogenes*, simply and efficiently cleaves a precise DNA target sequence that is complementary to the accompanying sgRNA (15). Cas9-mediated DSBs are repaired by the cell through non-homologous end joining, an innate, yet imprecise process that introduces insertions or deletions (indels) in the target DNA that lead to frameshift mutations in the corresponding protein sequence (16). Alternatively, DSBs can be mended through homology directed repair, which requires a donor DNA template with flanking homology arms to guide the precise, indel-free repair (17).

Successful CRISPR editing of the target DNA sequence can be validated by several methods (18). Publicly available bioinformatics platforms can be employed to estimate CRISPR editing efficiency using sequence decomposition. Briefly, control and CRISPR-edited DNA chromatogram traces are uploaded to the platform, which then identifies all possible indels for the control trace and determines the relative abundance of those indels in the mixed trace of the edited sample (19, 20). CRISPR editing efficiency can also be estimated by enzymatic detection of base pair mismatches. Control and CRISPR-edited DNA fragments are PCR amplified around the predicted cut sites, and the resulting DNA duplexes are denatured and randomly re-annealed to form heteroduplex DNA. A mismatch-identifying enzyme (e.g., T7 endonuclease 1; T7E1) recognizes and cleaves the CRISPR-generated base pair mismatches, which are detected by gel electrophoresis and quantified by band densitometry (21, 22). Alternatively, targeted next-generation sequencing (NGS) can be used to assess CRISPR-mediated indel frequencies and subsequent editing efficiencies (18).

### Advantages and Limitations of CRISPR

Unlike previous gene editing approaches such as homologous recombination, Cre-Lox, zinc-finger nucleases (ZFNs), or TALENs, CRISPR-Cas systems offer considerable advantages including improved target specificity, throughput, ease of use, and editing efficiency and precision (23, 24). CRISPR-Cas9 target specificity is determined by the 20 nucleotide sgRNA and a proximal protospacer adjacent motif (PAM) sequence (“NGG” for Cas9), which confers DNA target recognition by Cas9 (25). Multiple genomic sites may be targeted simultaneously with additional sgRNAs, which, together with Cas9 nuclease, are delivered to cells *in vitro* using traditional approaches such as electroporation or lipid-based transfection (15). CRISPR-Cas9 editing efficiencies may exceed 90% under ideal experimental conditions, though average efficiency may be ~40–50% at canonical NGG-adjacent target loci (26). The mean editing efficiency of CRISPR-Cas9 has been shown to be ~6 times greater than that of TALENs (15).

While CRISPR boasts enhanced target specificity compared to other gene editing technologies, the potential remains for off-target editing due to sufficient homology between the sgRNAs and off-target sequences in unintended genomic sites (27). To limit the possibility of off-target editing, guide design bioinformatics platforms predict off-targets by comparing the CRISPR sgRNA sequences with the whole genome of the species of interest (28). Alternatively, unbiased approaches for identifying CRISPR off-target sites, such as GUIDE-seq technology, can detect genome-wide DSBs for each sgRNA tested (29). The latest *in vitro* off-target prediction tools (e.g., CIRCLE-seq, SITE-seq, and Digenome-seq) allow for genome-wide as well as population-scale off-target profiling to account for genetic variants (30–32). If significant off-target editing is detected, wild-type CRISPR-Cas9 may be replaced with other CRISPR systems that offer improved on-target specificity with reduced off-target potential. These systems include Cas9 nickases that produce staggered DSBs from adjacent single-stranded cuts (33), as well as base or prime editors that convert specific nucleotide bases using catalytically impaired Cas9 fused to deaminase or reverse transcriptase (34–36). Alternatively, type V (e.g., Cas12) or type VI (e.g., Cas13) CRISPR systems may be utilized to produce staggered DNA DSBs or to directly target RNA, respectively (37, 38).

### CRISPR in the Clinic

To date, CRISPR has demonstrated promise in several therapeutic applications, which will undoubtedly guide the development of novel approaches for disease management and treatment. For example, CRISPR-Cas9 was used to delete a mutation in the *CEP290* gene that is responsible for Leber congenital amaurosis type 10 (LCA10), a severe form of retinal dystrophy that often stems from aberrant splicing due to a single point mutation (39, 40). *CEP290*-specific sgRNAs and Cas9 nuclease were delivered in viral vectors (adeno-associated virus, AAV) to non-human primate (NHP) photoreceptor cells using subretinal injection. CRISPR editing efficiencies of up to ~28% were observed for the treated NHPs, which exceeded the threshold of 10% functional rescue considered necessary to be clinically effective (39). Analogous CRISPR editing in human retinal explants followed by GUIDE-seq analysis found no evidence of off-target mutations. The strong preliminary data support the clinical investigation of the CRISPR-based therapeutic for treating *CEP290*-associated retinal disease (41).

Sickle cell disease (SCD) and transfusion-dependent β-thalassemia (TBT), two conditions of abnormal or insufficient erythrocytes resulting from mutations of the hemoglobin β subunit gene (*HBB*), were also targeted with CRISPR-Cas9 (42). Previously, single nucleotide polymorphisms (SNPs) in the *BCL11A* gene were found to correspond with increased fetal hemoglobin expression in adults, which subsequently correlated with reduced severity of SCD or TDT phenotypes (43, 44). Thus, to reactivate production of fetal hemoglobin, patients with SCD or TDT were infused with CRISPR-Cas9 edited hematopoietic stem and progenitor cells (HSPCs) that were mutated at the *BCL11A* locus. Preliminary data from two patients showed that the percentage of circulating erythrocytes expressing fetal hemoglobin increased from ~4 to >98% within 15 months of treatment (42). Regularly required disease-related transfusions were eliminated following treatment, and pre-clinical GUIDE-seq analyses found no evidence of off-target CRISPR editing (42).

The above applications underscore the value of CRISPR technology in tackling monogenic disorders, particularly those with relatively straightforward approaches for delivering treatments (e.g., direct subretinal injection or *ex vivo* blood cell editing). However, many CRISPR applications will require *in situ* editing of the relevant cells or tissues, which will ultimately necessitate more complex delivery methods or vehicles. Currently, *in vivo* delivery is predominantly limited to the use of viral vectors or lipid nanoparticles. Though viral vectors such as AAVs offer remarkable efficiency and specificity, the limited cargo capacity of the AAV genome inherently restricts the scope of potential applications (45). By contrast, delivering CRISPR reagents with nanoparticles may be constrained by potential toxicity concerns or inadequate targeting specificity (45). The current clinical applications of CRISPR editing, as well as the technological advances or limitations of those applications, will likely inform innovative treatments for many complex therapeutic targets including allergic disease.

## CRISPR Gene Editing of Allergens

### Editing the Major Cat Allergen, Fel d 1

Allergy to domestic cat (*Felis catus*, also known as *Felis domesticus*) affects 10–15% of adults and children, and may produce symptoms ranging in severity from rhinoconjunctivitis to asthma (46–48). Cat is the most common source of mammalian allergen, with high levels of the major cat allergen, Fel d 1, accumulating in house dust (10 → 1,000 μg/g dust) (49, 50). Roughly 95% of cat allergic patients produce IgE antibodies to Fel d 1, which accounts for 60–90% of total anti-cat IgE (51–55). Substantial exposure to Fel d 1 drives IgG4 antibody production in allergic and non-allergic individuals, and Fel d 1 is a prominent cause of Th2 immune responses (56). While several other cat allergens have been identified (e.g., Fel d 4), their allergenic and clinical significance has not been resolved (57, 58).

Fel d 1 is a tetrameric protein (35 kD) that is comprised of two heterodimers, each of which consists of two chains, chains 1 (70 AA, 8 kD) and 2 (92 AA, 10 kD) (59, 60). The genes, *CH1* and *CH2*, encoding chain 1 and chain 2, respectively, are situated in a span of ~10,000 base pairs in the genome. The structure of recombinant Fel d 1 (PDB 2EJN) indicates that the protein binds Ca^2+^ ions and contains internal hydrophobic cavities that may bind steroid ligands (61). Fel d 1 is a secretoglobin that is similar in structure to uteroglobin proteins, and is produced by cat salivary, lachrymal, sebaceous, and perianal glands (62–65). On average, kittens produce less Fel d 1 than adult cats, and females produce lower levels of Fel d 1 compared to males (66).

The precise biologic function of Fel d 1 is unknown, though studies of homologous proteins suggest the allergen may be involved in epithelium defense, immune regulation, or chemical communication (67–71). One recent study noted the sequence homology and common structural features between Fel d 1 and a defensive toxin secreted by the brachial glands of the slow loris primate (67). In another study, the binding properties of Fel d 1 were found to mirror those of mouse androgen-binding protein (ABP), a structural homolog of Fel d 1 that is secreted in mouse saliva and is involved in mate selection and chemical communication among mice (71–74).

Most treatment options for cat allergy sufferers merely address the allergic symptoms, which may have limited impact on patient health and quality of life. Immunotherapy for cat allergen using extracts or Fel d 1 peptides has been investigated, but consistent improvement for all patients has not been achieved (75, 76). Several recent approaches to cat allergy aim to reduce Fel d 1 exposure. One group introduced anti-Fel d 1 polyclonal egg IgY antibody into cat food to reduce the cats' salivary allergen levels (77, 78). The treated cats showed a 47% reduction in haircoat Fel d 1 compared to baseline (78). Alternatively, the immunization of cats with an anti-Fel d 1 vaccine resulted in a ~50% reduction in allergen detected in cat tear extracts and a ~30% decrease in allergic patient symptom severity (79, 80). The purported threshold at which nearly all cat allergic patients experience symptoms is 8 μg of Fel d 1 per gram of house dust (48). Therefore, a 50% reduction in Fel d 1 expression in a home with moderate levels of Fel d 1 (~100 μg/g dust) would likely have negligible clinical effects.

**Figure 1:**
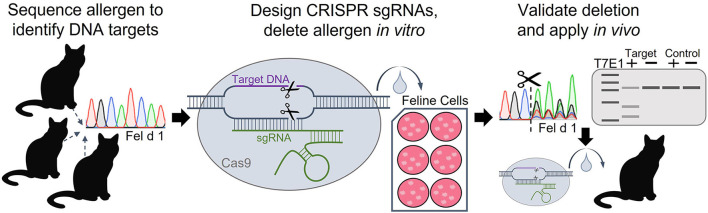
Workflow of experimental approach to delete the Fel d 1 genes using CRISPR-Cas9. Fel d 1 chains 1 and 2 were sequenced to identify conserved regions in the genes to target with CRISPR editing. Guide RNAs (sgRNAs) with sequences complementary to the conserved DNA target regions were designed and synthesized. The Fel d 1-specific sgRNAs and Cas9 nuclease were delivered to immortalized cat cells using lipid-based transfection. Successful *in vitro* editing, evaluated by DNA sequence decomposition and T7E1 (T7 endonuclease 1) mismatch detection, will guide future *in vivo* knockouts of Fel d 1.

Given that Fel d 1 is both an immunodominant allergen and a specific, well-defined genomic target, deleting Fel d 1 with CRISPR gene editing is a rational approach for tackling cat allergic disease. Recently, CRISPR technology was used to knockout the Fel d 1 genes *in vitro* (81–83). Genomic DNA was extracted from tissue samples of 50 domestic cats, and *CH1* and *CH2* were sequenced to identify conserved regions in the genes suitable for targeting with CRISPR (83). A panel of 10 sgRNAs targeted to either Fel d 1 chains 1 or 2 were evaluated. Each of the CRISPR sgRNAs along with Cas9 nuclease were delivered to immortalized feline kidney epithelial cells using lipid-based transfection. Fel d 1 gene knockout resulting from CRISPR-induced frameshift mutations was evaluated by DNA sequence decomposition or T7E1 mismatch detection for each sgRNA (Figure 1).

Sequence decomposition determined CRISPR editing efficiencies ranging from 5 to 55% for each of the 10 Fel d 1-specific sgRNAs, while T7E1 analysis found editing efficiencies of 5–45% (83). Analyses of several predicted potential off-target cleavage sites found no evidence of off-target CRISPR editing due to the Fel d 1-specific sgRNAs. Future studies aim to replicate the work in Fel d 1-expressing primary feline cells to confirm protein expression knockout and, eventually, to apply the work *in vivo* in cats. These preliminary *in vitro* data indicate that Fel d 1 is a viable target for gene deletion using CRISPR and provide the first step in creating Fel d 1-free cats. Targeting the allergen with CRISPR technology is expected to substantially benefit cat allergic individuals by effectively removing Fel d 1 at the source, and may serve as the critical step in determining the definitive, biologic function of the allergen.

### Editing Allergen Genes in Peanut

Allergy to peanut is one of the most severe food allergies and accounts for a significant proportion of food-induced allergic reactions that result in anaphylaxis (84, 85). The prevalence of peanut allergy among children in the US is ~2% but studies suggest this prevalence may be increasing (86, 87). While allergies to cow's milk or chicken egg proteins may resolve naturally during adolescence, allergy to peanut frequently persists into adulthood (88). Physical or chemical processes can be employed to reduce the allergenicity of peanuts and peanut products, however, avoidance or allergen immunotherapy [e.g., oral immunotherapy, Palforzia (89)] are recommended for sensitized individuals (90, 91). Several major peanut allergens have been identified including glycoprotein Ara h 2, which is recognized by IgE antibodies in more than 90% of peanut-allergic individuals (92, 93). Recently, peanut Ara h 2 was targeted using RNA interference (RNAi), a genetic engineering predecessor of CRISPR that knocks down gene expression at the mRNA level (94). An RNAi-expressing plasmid was delivered to peanut explants using *Agrobacterium*-mediated transformation, resulting in stable transgene integration in 44% of the plants. Seeds from the transgenic plants produced ~25% less Ara h 2 than control plants, and the IgE binding of peanut-allergic patient sera with the transgenic peanut samples was significantly reduced compared to wild type (94). The researchers propose transitioning from merely knocking down gene expression with RNAi to effectively deleting Ara h 2 and several other major peanut allergens using CRISPR (95).

### Editing Egg White Proteins in Chickens

Allergy to hen's egg is one of the more prevalent food allergies, affecting up to ~2% of young children in industrialized regions (96). Though egg allergy has been shown to naturally resolve in ~50% of allergic children, egg allergen avoidance remains challenging (97). The majority of the allergenic egg proteins from domestic chicken (*Gallus domesticus*) are found in egg whites, including ovalbumin (Gal d 2) and ovomucoid (Gal d 1) (98). Recently, the genes (*ovalbumin* and *ovomucoid*) that code for the egg white proteins were targeted with CRISPR editing. The genes were knocked out in cultured chicken primordial germ cells using CRISPR-Cas9 with sgRNA editing efficiencies ranging from 13 to >90% (99). The *ovomucoid*-free primordial germ cells were transplanted into chicken embryos, resulting in homozygous *ovomucoid* knockouts among the second generation of offspring (99). Analyses of several predicted potential off-target sites found no evidence of off-target CRISPR mutations. Though the allergenicity of eggs produced by the *ovomucoid* knockout chickens was not determined, the study demonstrates proof-of-principle for using CRISPR-Cas9 to eliminate the major egg allergen proteins and to ultimately produce hypoallergenic eggs.

### Editing Allergen Genes in Soybean

While soybean is an important food crop, several soy proteins are known to be major allergens. Allergies to soy-based protein formulas have been identified in ~0.5% of all children and up to 13% of children with other existing allergies (100). Given the value and abundance of soybean proteins that are increasingly used in food processing, gene editing offers a direct solution for developing hypoallergenic soybean products. Two soybean allergenic proteins include glycoprotein Gly m Bd 28 K (a Gly m 5 homolog) and oil-body-associated protein Gly m Bd 30 K (a cysteine protease), neither protein listed in the official WHO/IUIS Allergen Nomenclature database (101, 102). A recent study used CRISPR-Cas9 coupled with *Agrobacterium*-mediated transformation to simultaneously knockout the genes that code for these proteins in two varieties of soybean plants (103). Second and third generation soybean seeds exhibited indels at both target loci, including several deletions that produced frame-shift mutations and subsequently reduced protein expression and accumulation in the seeds (103). While Gly m Bd 30 K has been removed successfully from soy milk using pH-based protein fractionation (104), the CRISPR knockouts demonstrate proof-of-principle for the development of hypoallergenic soybean plants.

### Editing Allergen Genes in Wheat

Wheat is a staple food crop and a key element of human nutrition. Wheat grains are comprised of a broad spectrum of protein families, including the α-gliadin gluten proteins, which are primarily responsible for the development of celiac disease and gluten sensitivity (105). The α-gliadin genes contain several conserved stimulatory peptides including an immunodominant 33-mer peptide, which was targeted with CRISPR-Cas9 in polyploid bread and durum (pasta) wheat cultivars (106). Twenty-one transgenic wheat lines were produced, with CRISPR editing efficiencies of up to 75% detected (106). Gluten content or immunoreactivity of the edited lines was reduced by up to 85%, and no off-target mutations were observed at predicted potential off-target sites (106). Beyond gluten proteins, protective or metabolic proteins such as α-amylase/trypsin inhibitors (ATIs) contribute to the development of wheat allergies (107). CM3 and CM16, ATI subunit proteins shown to produce strong IgE reactivity, were targeted with CRISPR-Cas9 in durum wheat (108). Fourteen of 97 regenerated plants exhibited CRISPR edits in the *CM3* or *CM16* genes, which were evaluated by sequencing and biochemical analyses (108). Taken together, these studies demonstrate the value of high-throughput CRISPR editing for the development of novel wheat varieties with reduced immunogenic profiles. Though the polyploid nature of wheat poses the additional challenge of simultaneously targeting several alleles or gene copies to achieve a functional knockout, the enhanced efficiency and versatility of CRISPR systems will certainly improve the targeted editing of polyploid genomes compared to traditional breeding approaches (109).

### Editing β-Lactoglobulin in Cow's and Goat's Milk

Allergy to milk is the most common childhood food allergy, with an estimated 3% of infants experiencing adverse reactions to cow's milk proteins (110). The major cow's milk allergens include caseins and β-lactoglobulin (BLG, also known as Bos d 5), though several other minor allergen proteins have been identified (111). β-lactoglobulin, the primary component of milk whey proteins, is a particularly important allergen given its absence from human milk. Several groups have applied gene editing technology to produce BLG-free cows and goats. A *BLG* gene knockout cow generated using ZFN technology, a predecessor of CRISPR, produced BLG-free milk that resulted in significantly less IgE binding in cow's milk allergic individuals compared to wild type (112). Whole genome sequencing found no off-target effects due to the *BLG*-specific ZFN mRNA, while PCR confirmed that the *BLG* mutation is stably passed to offspring through germline transmission (112). Similarly, CRISPR-Cas9 was used to generate *BLG* knockout goats. Three *BLG*-specific CRISPR sgRNAs were co-injected with Cas9 mRNA into goat embryos, resulting in editing efficiencies of ~25% (113). The *BLG*-knockout goats produced significantly less BLG protein in milk, and no off-target editing was detected at predicted potential loci (113). These hypoallergenic milk studies highlight the value and therapeutic potential of applying genome editing technology to livestock for the benefit of human health.

## Summary

**Figure 2:**
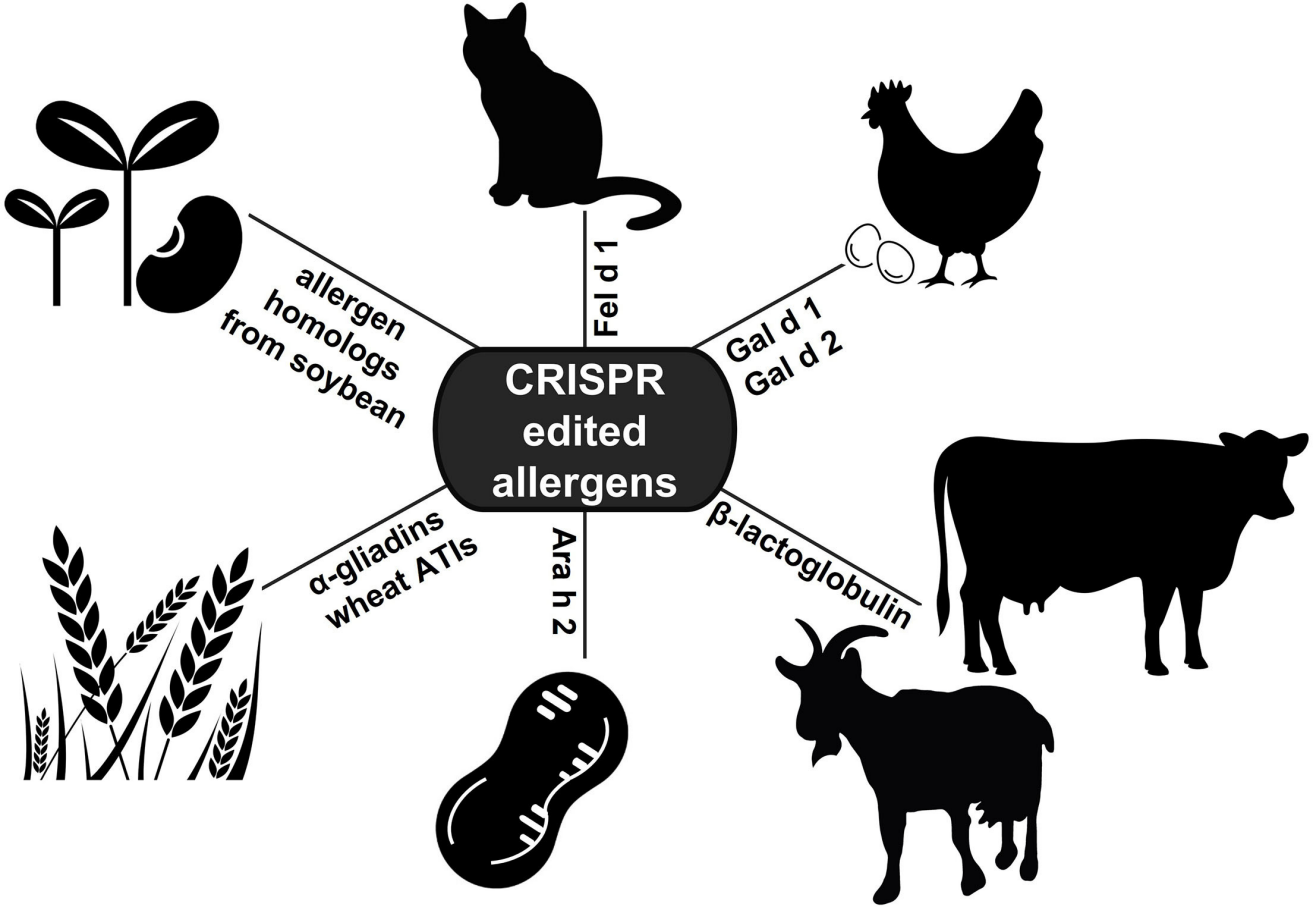
Applications of CRISPR editing in allergy research. To date, CRISPR technology has been applied to edit allergen genes in cat, hen's egg, soybean, wheat, peanut, and cow's and goat's milk (ATIs: α-amylase/trypsin inhibitors).

CRISPR editing has shown promise in numerous applications of allergy research (Figure 2). These studies demonstrate the value of the technology in improving our understanding of allergen proteins, and underscore the vast potential for CRISPR editing to provide better, alternative treatment options for allergic disease. The applications highlighted in this review illustrate how CRISPR may be used to determine allergen protein function, engineer hypoallergenic foods, or develop allergen-free animals. In the future, CRISPR technology could also be employed for the identification of novel allergens, or for modifying the immune response directly as an approach to prevent recognition of allergen proteins. Moving forward, comprehensive analyses of allergen protein sequences, structures, or antibody binding sites will be invaluable for identifying essential functional domains or conserved sequences to target with CRISPR deletion. Additionally, further development of methods for the targeted delivery of CRISPR reagents to specific cells or tissues *in vivo* will prove vital for successfully editing the allergen genes in adult animals. While the value of CRISPR gene editing as a revolutionary therapeutic approach has only recently been established, the technology is poised to transform the management and treatment of allergic disease.

## Author Contributions

NB wrote the manuscript. All of the authors critically reviewed and revised the manuscript.

## Funding

This study received funding from INDOOR Biotechnologies Inc.

## Conflict of Interest

MC is a co-owner and founder of INDOOR Biotechnologies Inc. NB and AP are employees of INDOOR Biotechnologies Inc.

## Publisher's Note

All claims expressed in this article are solely those of the authors and do not necessarily represent those of their affiliated organizations, or those of the publisher, the editors and the reviewers. Any product that may be evaluated in this article, or claim that may be made by its manufacturer, is not guaranteed or endorsed by the publisher.
